# Dual public health crises: the overlap of drug overdose and firearm injury in Indianapolis, Indiana, 2018–2020

**DOI:** 10.1186/s40621-022-00383-9

**Published:** 2022-07-03

**Authors:** Lauren A. Magee, Bradley Ray, Philip Huynh, Daniel O’Donnell, Megan L. Ranney

**Affiliations:** 1 O’Neill School of Public and Environmental Affairs, Indiana University Purdue University Indianapolis, 801 W Michigan St, Rm 4058, Indianapolis, IN 46202 USA; 2 Division for Applied Justice Research, RTI International, 3040 Cornwallis Road, Research Triangle Park, Durham, NC 27709 USA; 3 Center for Behavioral Health and Justice, Wayne State University School of Social Work, 5201 Cass Avenue, Prentis, Suite 226, Detroit, MI 48202 USA; 4 Indiana University School of Medicine, Indianapolis Emergency Medical Services, 3930 Georgetown Rd., Indianapolis, IN 46254 USA; 5 School of Public Health and Alpert School of Medicine, Brown University, 121 S Main St, Providence, RI 02903 USA

**Keywords:** Drug overdose, Firearm injuries, Neighborhoods, Public health

## Abstract

**Background:**

Drug overdose and firearm injury are two of the United States (US) most unrelenting public health crises, both of which have been compounded by the COVID-19 pandemic. Programs and policies typically focus on each epidemic, alone, which may produce less efficient interventions if overlap does exist. The objective is to examine whether drug overdose correlates with and is associated with firearm injury at the census tract level while controlling for neighborhood characteristics.

**Methods:**

An ecological study of census tracts in Indianapolis, Indiana from 2018 to 2020. Population rates per 100,000 and census tracts with the highest overlap of overdose and firearm injury were identified based on spatial clusters. Bivariate association between census tract characteristic and drug overdose and firearm violence rate within spatial clusters. Zero-inflated negative binominal regression was used to estimate if the drug overdose activity is associated with higher future firearm injury.

**Results:**

In high overdose—high firearm injury census tracts, rates of firearm injury and drug overdose are two times higher compared to city wide rates. Indicators of structural disadvantage and structural racism are higher in high overdose—high firearm injury census tracts compared to city-wide averages. Drug overdoses are associated with higher rates of firearm injury in the following year (IRR: 1.004, 95% CI 1.001, 1.007, *p* < 0.05), adjusting for census tract characteristics and spatial dependence.

**Conclusions:**

Drug overdose and firearm injury co-spatially concentrate within census tracts. Moreover, drug overdoses are associated with future firearm injury. Interventions to reduce firearm injuries and drug overdoses should be a co-response in high drug overdose—high firearm injury communities.

## Introduction

Drug overdose and firearm injury are two of the United States (US) most unrelenting public health crises, both of which have been compounded by the COVID-19 pandemic (Faust et al. 2021). These two epidemics are often thought of and treated as separate public health crises, despite common underlying drivers (e.g., poverty, hopelessness, structural racism, criminalization of drug use, co-existing problem behaviors). A considerable amount of local, state, and federal resources has focused on each epidemic, alone, examining the relationship between the two may allow identification of more efficient and appropriate policies and programs can address these dual public health crises.

Overdose deaths continue to increase across North America largely driven by opioids. In recent years, the underlying substances have shifted from prescription opioid analgesic medications to illicitly produced fentanyl, a synthetic opioid 50 to 100 times more potent than morphine, has contaminated much of the drug supply, particularly heroin (Ciccarone 2021). Criminalization of opioids among persons who are already dependent has increased overdose deaths (Davis et al. 2019), and the introduction of fentanyl into the drug market coincided with dramatic population shifts as overdose deaths among Black Americans now outpace Whites (Furr-Holden et al. 2021). While historical trends show opioids and stimulants alternating in periods of use as most common overdose, current patterns suggest polydrug co-use among these substances along with cutting fentanyl into other illicit substances, including heroin (Strickland et al. 2019), sparking a fourth wave of the drug epidemic with overdoses involving both opioids and stimulants.

Overdose events geographically cluster and likewise (Marshall et al. 2020; Mohler et al. 2021), firearm injury events cluster in (Branas et al. 2017; Braga et al. 2009) relatively small areas of cities (i.e., hot spots) (Magee 2020; Koper et al. 2015). Thus, research on firearm injury, including homicide, has consistently focused on neighborhood conditions which include economic disadvantage (including deprivation and a lack of resources), residential instability, racial segregation, and the prevalence of firearm ownership, aggregated to neighborhood levels (i.e., census tract, county level), as causal mechanisms (Hepburn and Hemenway 2004; Sampson et al. 1997). As firearm injuries in the USA increase to rates not seen in six decades, researchers and policymakers aim to identify how these and other mechanisms might contribute.

Co-existing individual-level risk factors do exist between opioids and firearm injuries. Opioid users have higher rates of gun involvement than alcohol users (Stein et al. 2018) and youth who report opioid use also report weapon carriage (Pontes and Pontes 2021), both demonstrate co-existing problem behaviors can lead to overdose and firearm injuries. Community-level conditions of the illicit drug market, however, are more predictive of violence than specific individual drug effects as researchers have long noted the association between drugs and violence largely stems from the conditions of the illicit market rather than specific drug effects (Goldstein 1985; Resignato 2000; Donohue and Levitt 1998). Illicit drug markets have been described as “stateless” social spaces where violence is used to resolve conflicts because formal social control (police and courts) is unavailable. When drug markets shift, as they have done through multiple waves of the overdose epidemic (from prescription to illicit opioids and then to illicit stimulants), there are increased conflicts over price, purity, and other purchasing terms which research suggests is likely to result in violence (Blumstein and Rosenfeld 1997); however, few studies have looked at the overlap between violence from firearm injury and overdose to capture drug market activity in this recent crisis.

In a national level study from 1999 to 2015, race-specific overdose was positively associated with race-specific homicide rates in the following year when adjusting for county level context (Rosenfeld et al. 2021), and the increase in overdose was correlated with higher homicide rates over time (Rosenfeld and Roth 2021). These studies were limited to national overdose data and only looked at opioid-related events to measure the demand of illegal drug markets, which are known to be undercounted in many parts of the USA (Ruhm 2016). In rural counties, overdoses were associated with firearm-related hospital admissions (Dittmer et al. 2021), and drug-related fatal overdoses overlapped with higher rates of firearm suicide at the county level (Kalesan et al. 2020). These studies were not situated to determine causality or only examined rural counties. Despite limitations, these studies provide preliminary evidence drug overdose and firearm injury overlap. Further investigation is warranted to clarify whether changes in individual or structural policies and programs are warranted.

The objective of this study is to examine whether drug overdose, as an indicator of drug market activity, spatially correlates with and is associated with future firearm injury and census tract characteristics. This study is positioned to answer these questions because we have reliable data on the full spectrum of outcome measures (fatal and nonfatal) firearm injuries and on the neighborhood characteristics. The characteristics of overdose have changed over time, and we have the ability to look at both fatal and nonfatal overdose, and Indianapolis is similar to many other urban and semi-urban areas currently experiencing increases in both overdoses and firearm injuries. Indianapolis is the largest county (Marion) in Indiana and 12th largest city in the USA. In 2019, Indianapolis had the 18th highest overdose mortality rate (25.6 per 100,000 population) in the USA and accounted for more than 20% of all the fatal overdoses in the state. In 2020, Indianapolis was the 11th most violent city in the USA, primarily due to firearm injury.

## Methods

In an ecological study, we examine whether overdose is associated with firearm injury when adjusting for several census tract characteristics leveraging a unique dataset of drug overdoses and firearm injuries between January 1, 2018, and December 31, 2020. The period allows for pre-pandemic and peri-pandemic, as well to examine if drug overdoses spatially overlap and are associated with firearm injury. We utilized the census tract as our unit of analysis to assess variation within and across the county. This study was approved and considered exempt by Indiana University institutional review board.

### Data and measures

Unlike prior studies, we use an expanded definition of firearm injury and overdose by including nonfatal events to capture drug market activity in the community. N*onfatal overdose* events were identified through Indianapolis Emergency Medical Services (EMS) data where the chief complaint, secondary complaint, and mechanism of injury identified overdose and/or naloxone (the opioid antagonist) was administered (*n* = 16,178) (Glober et al. 2020; Ray et al. 2018). Events included the date and location of the incident for geocoding; 2.59% (*n* = 419) of events were unable to be geocoded and were removed. To identify *fatal overdose* events, we used local death investigation records and corresponding postmortem toxicology results all accidental drug overdose deaths (International Classification of Diseases (ICD) codes X40-X44) which contain information on the time and location of the death along with whether illicit opioids (heroin or fentanyl) and stimulants (methamphetamine or cocaine) were identified. The fatal overdose data range from 2018 through 2020 (*n* = 822), with 6% excluded that did not geocode.

Data on firearm injury include homicides and nonfatal shootings; *firearm homicides* (*n* = 438) were obtained from the *Indianapolis Star* (newspaper) which collects publicly available data on all criminal homicide investigations conducted by the Indianapolis Metropolitan Police Department (IMPD) (IndyStar. 2019). The number of victims was verified with official police records. Data include information on date of incident, method of death, weapon, victim race, and incident location. All victims with firearm listed as the method of death were included. *Nonfatal firearm injury* data (*n* = 1715), defined as an injury caused by a projectile weapon and powder discharge during a criminal assault, were obtained from IMPD, as police data are more complete than clinical records when compared at the incident level (Magee et al. 2021). Information available includes incident date, incident location, and victim race-ethnicity. Suicides and police-involved shootings were excluded due to incomplete data capture and availability.

For both overdose and firearm injury, we examined the fatal and nonfatal events separately but also combined these events for a composite measure of overdose and firearm injury. To measure *census tract-level characteristics*, we used US census data and selected variables of interest based on prior firearm injury and drug-related mortality research (Sampson et al. 1997; Rosenfeld et al. 2021; Dittmer et al. 2021; Mars et al. 2015). These characteristics include the percent of Black residents, percent of Hispanic residents, percent of residents living in poverty, percent of female-headed single households, percent unemployed, percent public assistance, percent male population between 15 and 24 years of age, percent of renters, and the total population. The number of abandoned homes was obtained from the Indianapolis open data portal (https://data.indy.gov/) and contains the street address of all abandoned residential homes. We gathered the number of residential homes from the US Census and calculated the percent of abandoned homes within each census tract. All census tract-level characteristics were included as continuous measures. Using factor analysis, the percent of residents living in poverty, percent of female-headed single households, percent unemployed, and percent on public assistance were combined as a measure of structural disadvantage in multivariate models.

Emergency management services (EMS) activity was also included in the analyses. EMS activity was defined as all dispatch runs excluding overdoses, and the EMS activity measure was included as a *z*-score (Johnson and Roman 2022). A spatially lagged variable on the dependent variable was included to account for positive spatial autocorrelation. The variable was created using first-order Queen’s contiguity spatial weights matrix using Geoda (Magee 2020).

### Statistical analysis

Our analysis begins with trends of postmortem toxicology results to illustrate changes in the drug market during the study period and descriptive statistics on overdose and firearm injury, along with all census tract characteristics. To examine the geographic patterns and association of overdose and firearm injury, we created choropleth maps using natural breaks. To assess the spatial association between overdose and firearm injury, we conducted a bivariate Moran’s I using a first-order Queen’s contiguity spatial weights matrix using Geoda software (Magee 2020), which classifies each census tract based on a weighted average of adjoining census tracts, a given value, and a spatial lag term. The Moran’s scatter plot provides spatial clusters based on four categories, high-high, low-low, low–high, and high-low for those that are statistically significant. The census tracts with higher means have “high” values, and census tracts below the mean have “low” values. Next, we examined the bivariate association between each census tract characteristic and the overdose rate and firearm violence rate per the four categories of spatial clusters defined above using paired *t*-tests. The population rate per 100,000 was calculated as the number of events over the total population within a census tract. Lastly, we build on the descriptive and geospatial analysis with multivariate models to test if overdoses to capture drug market activity is associated with higher rates of future firearm injury. Due to tracts with zero counts, skewness, and over-dispersion in each census tract, we performed zero-inflated negative binominal regression models to estimate incident rate ratios (IRR). Total census tract population was used to estimate the excess zeros in the models. We estimate separate change models for overdose in 2018 and 2019 and firearm injury in 2019 (pre-pandemic) and 2020 (peri-pandemic) while adjusting for census tract characteristics and spatial dependence.

## Results

### Changing drug market

**Fig. 1:**
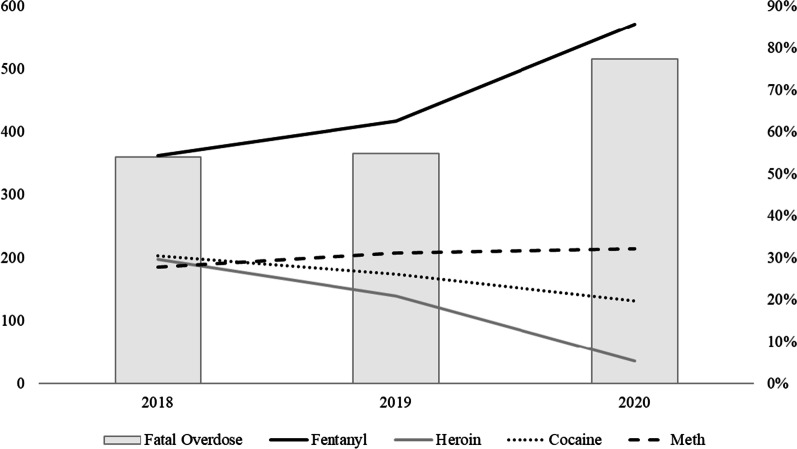
Toxicology results among overdose deaths as a reflection of the changing drug market. Notes: Bars represent the total number of accidental drug overdose deaths each year and lines represent the proportion of deaths where that substance was detected in toxicology results. Detection of substances is not mutually exclusive as most overdose deaths contain multiple substances

From 2018 to 2020, the composite overdose rate was 621.9 per 100,000 population and the composite firearm injury rate was 83.9 per 100,000 population. Compared to 2019, 2020 saw an increase in both drug overdoses (577.9/100,000 vs. 734.5/100,000) and firearm injury (105.1/100,000 vs. 74.9/100,000). Fentanyl was the predominate drug contributing to overdose deaths between 2018 and 2020, representing 85.6% of overdose in 2020 (Fig. 1).

### Spatial clustering of overdose and firearm injury

**Fig. 2:**
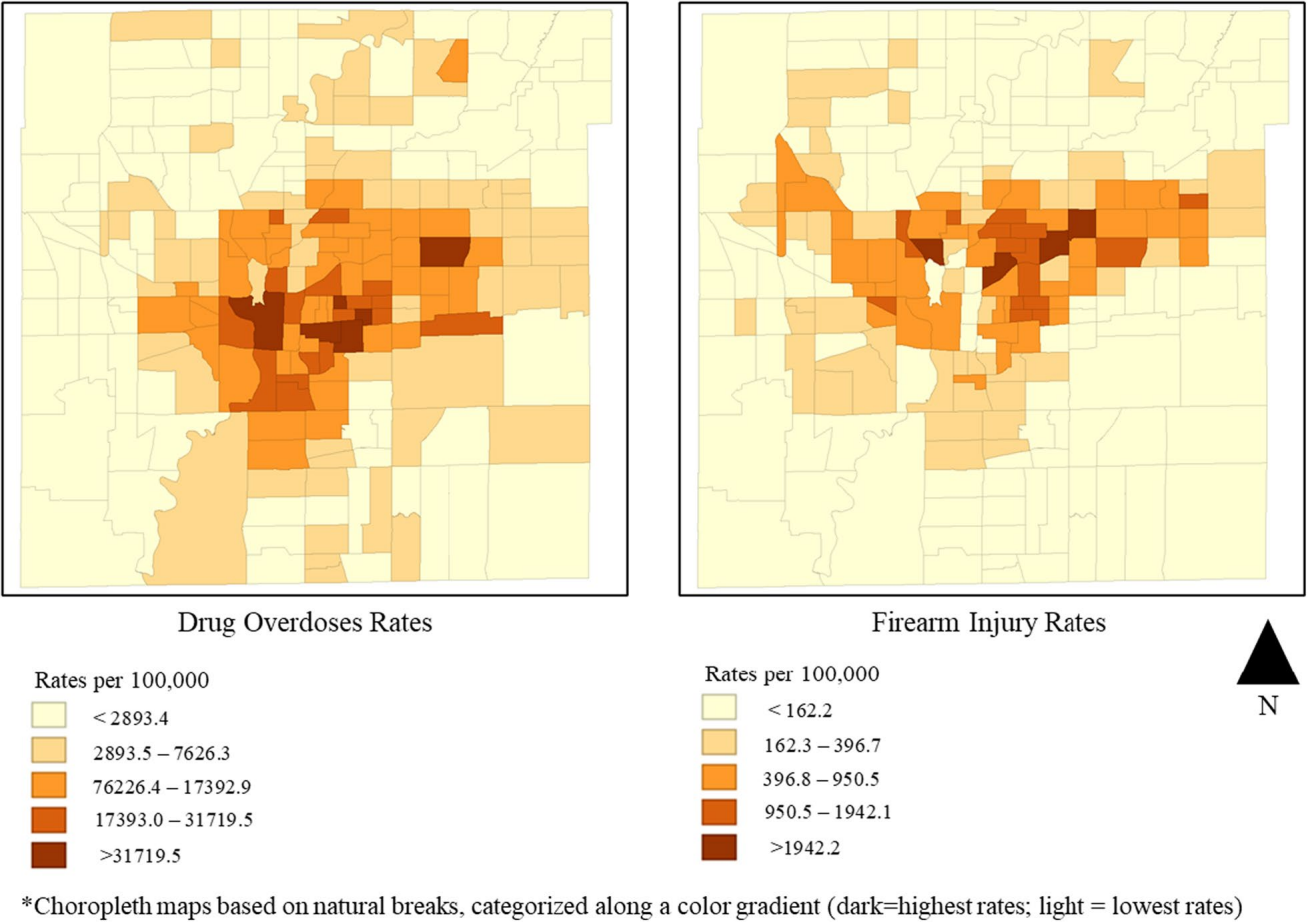
Drug overdose and firearm injury rates, Indianapolis, Indiana, 2018–2020

**Fig. 3:**
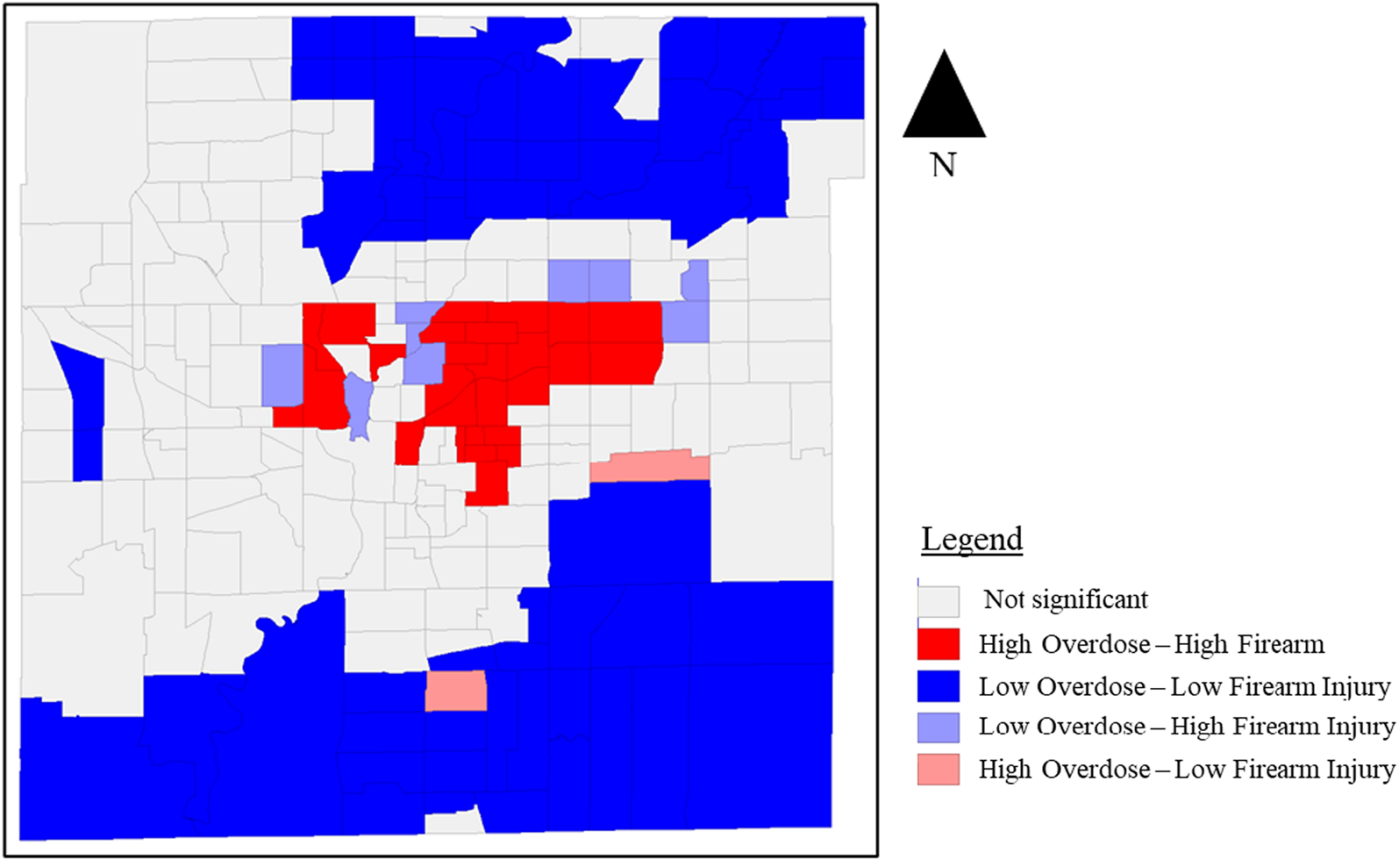
Bivariate spatial clusters of drug overdoses and firearm injury

Choropleth maps display clusters and a statistically significant spatial autocorrelation (Moran’s I: 0.372; *p* < 0.001) between overdose and firearm injury rates across census tracts (Fig. 2). There were 27 census tracts with statistically significant spatial clusters of high overdose and high firearm injury, nine census tracts with low overdose and high firearm injury, two census tracts with high overdose and low firearm injury, and 57 census tracts with low overdose and low firearm injury (Fig. 3). Census tracts with the highest overlaps of high overdose and high firearm injury experience a composite overdose rate of 18,340.5 per 100,000 and a composite firearm injury rate of 2,453.6 per 100,000; compared to city wide rates of 100.2/100,000 and 69.2/100,000, respectively (Table 1).

**Table 1:** Characteristics of spatial clusters of drug overdose and firearm injury rates

	City wide	High firearm injury–high overdose	High firearm injury–low overdose	High overdose–low firearm injury	Low firearm injury–low overdose
	Mean (SD)	Mean (SD)	Mean (SD)	Mean (SD)	Mean (SD)
*Rates per 100,000 population*	*N* = 216	*n* = 27	*n* = 9	*n* = 2	*n* = 53
Firearm homicide	69.2 (118.6)	2453.6 (221.5)	118.0 (93.9)	19.6 (27.7)	10.9 (22.7)
Nonfatal firearm injury	245.6 (319.4)	786.9 (402.7)	320.6 (161.1)	74.5 (5.50)	33.9 (46.1)
Composite firearm injury	314.8 (418.6)	1030.3 (564.9)	438.7 (201.7)	94.1 (33.2)	44.7 (63.3)
Fatal drug-related overdose	100.2 (98.4)	252.7 (141.4)	83.9 (55.5)	172.4 (144.0)	44.6 (40.0)
Nonfatal drug-related overdose	7178.9 (8105.6)	6029.3 (3128.5)	1744.1 (363.8)	4648.4 (3179.3)	800.2 (522.7)
Composite drug-related overdose	7279.1 (8171.3)	18,340.5 (9421.7)	5316.1 (1114.7)	14,117.7 (9682.1)	2445.2 (1589.5)
*Neighborhood characteristics*					
% Poverty	**28.2 (18.2)**	**49.1 (15.2)**	**35.7 (10.2)**	23.6 (4.90)	**13.4 (9.69)**
% Single female-headed house	**9.36 (8.97)**	**17.5 (9.16)**	**11.1 (6.20)**	2.32 (1.51)	**3.87 (4.76)**
% Unemployed	**4.65 (3.37)**	**8.37 (3.41)**	**8.17 (4.70)**	3.07 (0.91)	**2.37 (1.18)**
% Public assistance	**1.99 (1.83)**	**1.87 (1.15)**	**1.72 (1.19)**	4.73 (2.84)	**1.05 (1.13)**
% Black	**30.5 (24.9)**	**58.2 (24.1)**	**62.7 (14.4)**	2.84 (2.47)	**11.1 (11.6)**
% Hispanic	**10.2 (9.10)**	**12.6 (9.02)**	**7.94 (7.06)**	3.22 (0.40)	**5.03 (4.48)**
% Male population 15–24	**13.1 (6.26)**	**12.9 (5.81)**	**14.3 (6.62)**	10.3 (4.09)	**11.9 (4.78)**
% Renters	**40.4 (18.2)**	**46.1 (9.19)**	**45.6 (17.7)**	25.5 (1.20)	32.8 (21.1)*
% Abandoned homes	**3.51 (5.57)**	**13.0 (6.22)**	**5.28 (5.10)**	0.51 (0.45)	**0.14 (0.28)**

Spatial clusters based on Moran’s I scatter plot of statistically significant census tracts

Bolded values indicate *p* < 0.05 of paired *t*-tests of neighborhood characteristic and composite overdose and firearm injury rate

*Bolded values indicate *p* < 0.05 of paired *t*-tests of neighborhood characteristic composite overdose rate only

In high overdose and high firearm injury census tracts, indicators of structural disadvantage are higher compared to city-wide averages; 49% of residents live below the poverty level, 17.5% are single female-headed households, 8.37% are unemployed, 58.2% of residents identify as Black, 46.0% are renters and 13.0% of residences are abandoned properties. Similar census tract characteristics are observed in high firearm injury and low overdose tracts, but rates of poverty (35.7%) and abandoned homes are lower (5.28) compared to high-high census tracts.

### Multivariate results

In both 2018–2019 and in 2019–2020, prior year drug overdoses (2018: IRR: 1.005, 95% confidence interval (CI): 1.001, 1.009, *p* < 0.05 and 2019:IRR: 1.004, 95% CI 1.001, 1.007, *p* < 0.05) are associated with higher rates of firearm injury (composite: both fatal and nonfatal) in the following year, adjusting for census tract characteristics, spatial lag, and population per 100,000 (Table 2). Racial and ethnic characteristics of a census tract as well as structural disadvantage are also associated with the next year’s firearm injury rates.

**Table 2:** Drug Overdoses associated with Firearm Injury, by year and neighborhood characteristics

	Model 1	Model 2
	Composite firearm injury, 2019	Composite firearm injury, 2020
	IRR (95% CI)	IRR (95% CI)
*Neighborhood characteristics*		
Drug overdoses, 2018	**1.005 (1.001, 1.009)**	–
Drug overdoses, 2019	–	**1.004 (1.001, 1.007)**
Structural Disadvantage	1.192 (0.961, 1.479)	**1.263 (1.026, 1.556)**
% Black	**1.018 (1.011, 1.026)**	**1.018 (1.011, 1.025)**
% Hispanic	**1.023 (1.009, 1.037)**	**1.020 (1.007, 1.032)**
% Male Population 15–24	1.009 (0.991, 1.027)	0.999 (0.983, 1.016)
% Renters	1.001 (0.992, 1.010)	1.004 (0.995, 1.014)
% Abandoned Homes	1.018 (0.994, 1.043)	0.998 (0.972, 1.024)
EMS Activity	1.062 (0.722, 1.562)	0.965 (0.711, 1.310)
Spatial Lag	1.033 (0.985, 1.082)	**1.054 (1.000, 1.111)**
AIC	796.31	924.75
BIC	839.95	968.38

Bolded values indicate *p* < 0.05, IRR: incident rate ratio, CI: confidence interval

## Discussion

This study is groundbreaking in demonstrating the degree of correlation between two persistent and escalating social problems in the USA: drug overdose and firearm injury in a city with highly reliable data on both. Major findings include overdose is more prevalent than firearm injury, even with firearm injury at unprecedented levels (Faust et al. 2021). Twelve percent of the census tracts in the county had high overlap of both overdose and firearm injury and were more likely to be in structurally disadvantaged communities with residents living in poverty. Surprisingly, overdose and firearm injury were so highly concentrated in these census tracts rates of firearm injury and drug overdose is two times higher compared to city wide rates. Our study also shows census tract overdose rates that are associated with future firearm injury and death. Overall, our findings indicate; (1) the importance of addressing the two epidemics simultaneously, and (2) the association of drug overdoses, structural disadvantage, structural racism, and firearm injury and death.

Our findings indicate there are spatial clusters of high overdose and high firearm injury rates and overdose rates are positively associated with future firearm injury rates, which align with prior research (Rosenfeld et al. 2021; Rosenfeld and Roth 2021). Our study also identifies common underlying neighborhood factors associated with overdoses and firearm injury, particularly for predominantly Black communities with high levels of structural disadvantage and racism. Our findings extend prior research to demonstrate within county variation and spatial clustering among census tracts indicating the importance of examining both overdoses and firearm injury at levels of geography smaller than a county. Similar to our findings, more than 300 counties in the USA bear a disproportionate burden of firearm suicide and overdose, were spatially next to urban areas, and are considered “diseases of despair,” due to both being main contributors to the decline in life expectancy of Americans (Kalesan et al. 2020). Given our findings suggest similar overlap between overdose and fatal and nonfatal firearm injuries, we need to consider all firearm injuries as a marker of "diseases of despair" and overdose and firearm prevention strategies should be combined and not thought of, or treated, as separate public health crises. For example, police and public health approaches to implement co-response efforts and empower overlapped “hot spot” communities to deploy harm reduction strategies like naloxone distribution, drug checking services, and overdose prevention sites, to reduce the immediate harms while also focusing on longer term community interventions that improve collective efficacy (Sampson et al. 1997; Zibbell et al. 2021). For instance, research consistently suggests community greening projects, which seek to foster resident involvement in beautifying vacant lots decreases community violence (Kondo et al. 2016; Pizarro et al. 2020). Abandoned homes were not independently associated with firearm injuries in our study, but prior work suggests an association between firearm injury and abandoned homes (Magee 2020).

Although this descriptive study cannot determine causality, research into increasing overdose rates points toward an illicit drug supply, particularly heroin, that is being contaminated or replaced with fentanyl (Ciccarone 2021). Thus, as the drug supply changes there are increased conflicts over price, purity, and other purchasing terms that are addressed through firearm injury. Our findings indicate the importance of the spatial clustering of drug overdose and firearm injury, and it is plausible the high rates of drug overdose are associated with the illicit drug market. This notion aligns with prior research that finds proximity to drug markets, and drug market activities are associated with higher rates of shootings following the onset of the COVID-19 pandemic (Johnson and Roman 2022). Research also demonstrates a perceived need to have a firearm for self-protection in stateless markets (Donohue and Levitt 1998), and co-existing risk factors among opioid users and firearm injuries exist (Stein et al. 2018; Pontes and Pontes 2021) therefore increasing the chance for firearm injury. Future examination into the illicit drug market, drug overdose, and firearm injuries is warranted to identify appropriate policies and programs at the local, state, and federal level.

## Limitations

This study does not allow for individual level assessment; however, ecological studies examine which neighborhoods are most at risk (Zeoli et al. 2019). Although we only examine one jurisdiction, our findings demonstrate the need to disaggregate below the county level. Overdose and firearm injury rates may be overestimates in census tracts with low residential population. Our data were not designed for measuring causal effects, we also did not examine polydrug overdoses, shooting motives, different temporal lag times, and other potential confounders, but these are clear directions for future research. We are unable to determine if the same individuals overdosing were involved in firearm injury or if these are simply co-occurring events in census tracts. Future research should link individual level data to examine both the individual and area-level effects. Lastly, overdoses may be an imperfect measure of illicit drug markets, future studies should further explore police drug seizures as measures of illicit drug markets, as well as the relationship between changing drug markets and firearm injury. Despite these limitations, we provide a major advancement in our understanding of these phenomena by using multiple localized data sources to examine fatal and nonfatal overdose and firearm injury.

## Conclusion

This study found drug overdose and firearm injury events are highly concentrated in similar geographic spaces, particularly among marginalized populations. Findings also suggest drug overdose events, which may capture drug market activity, are associated with rising rates of firearm violence. These findings speak to the need for a combined public health approach to drug overdose and firearm injury, not individual plans for each epidemic, and that intervention strategies should aim to empower and repair the harms to disadvantaged populations (Bluthenthal 2021).

## Data Availability

Data were acquired from third parties and maybe available from the authors or third-party upon reasonable request.

## Abbreviations

US: United States
EMS: Emergency medical services
ICD: International classification of diseases
IMPD: Indianapolis Metropolitan Police Department
IRR: Incident Rate Ratio
CI: Confidence intervals
